# SARS-CoV-2 infection and profound hearing loss: much more than a coincidence

**DOI:** 10.31744/einstein_journal/2026AO1126

**Published:** 2026-01-28

**Authors:** Pedro Luiz Mangabeira Albernaz, Sady Selaimen da Costa, Vinícius Oliveira Nitz

**Affiliations:** 1 Hospital Albert Einstein São Paulo SP Brazil Hospital Albert Einstein, São Paulo, SP, Brazil.; 2 Universidade Federal de São Paulo São Paulo SP Brazil Universidade Federal de São Paulo, São Paulo, SP, Brazil.; 3 Universidade Federal do Rio Grande do Sul Hospital de Clínicas de Porto Alegre Porto Alegre RS Brazil Hospital de Clínicas de Porto Alegre, Universidade Federal do Rio Grande do Sul, Porto Alegre, RS, Brazil.

**Keywords:** COVID-19, Hearing tests, Hearing loss, sensorineural, Cochlear implants, Quality of life

## Abstract

**Objective:**

To describe a series of cases in which severe acute respiratory (SARS-CoV-2) infection was associated with profound sensorineural hearing loss. Our data provides extensive evidence that COVID-19 infections can cause sensorineural hearing losses.

**Methods:**

This study included seven patients with profound sensorineural hearing loss caused by COVID-19. Patients were subjected to clinical evaluation, COVID-19 symptoms, and audiological tests.

**Results:**

Brief clinical histories and audiological tests were performed. The current condition of the patients was also considered. Theories of the possible causes of inner ear changes are also discussed.

**Conclusion:**

This study demonstrates that the world continues to grapple with the far-reaching consequences of COVID-19, and it becomes evident that the impact extends beyond the immediate respiratory distress.

## In Brief

Hearing loss after SARS-CoV-2 infection is not a very common complication. However, the case presented here experienced severe hearing loss. Although bilateral simultaneous sensorineural hearing loss is very rare, it is the most common type of post-COVID hearing loss. Some patients may be assisted by wearing hearing aids; however, many have profound hearing loss and require cochlear implants. This study provides evidence that hearing loss may be a serious complication of post-COVID infection.

## Highlights

■ This study describes a series of cases in which SARS-CoV-2 infection is associated with profound sensorineural hearing loss.
■ COVID-19 infections can indeed cause sensorineural hearing loss.
■ Seven patients with profound sensorineural hearing loss due to COVID-19 were clinically evaluated and underwent audiological testing.
■ This study highlights that the consequences of COVID-19 extend beyond respiratory symptoms and affect other systems, such as the inner ear.

## Graphical abstract

**Figure:**
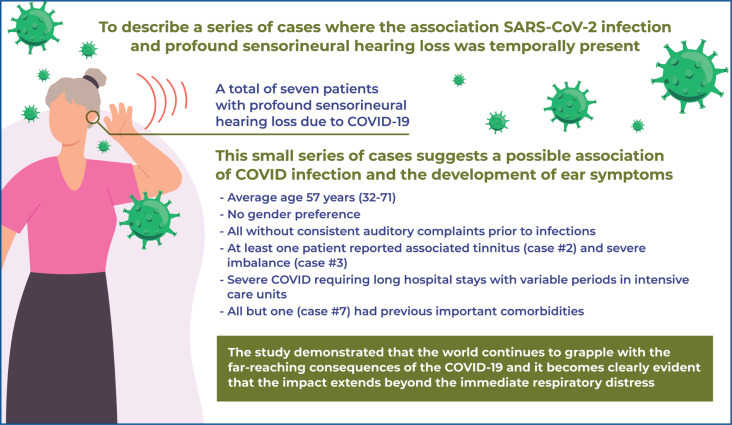

## INTRODUCTION

The global pandemic caused by the severe acute respiratory syndrome coronavirus 2 (SARS-CoV-2) virus, which initially emerged in Wuhan, China, quickly broke demographic barriers, spreading across the planet, leaving a trail that accounted for millions of deaths and countless, disabling consequences.

The disease is characterized by a series of signs and symptoms that can be conceptually grouped into typical (related to the upper airway) and atypical (other organs and systems).

The typical clinical picture based on confirmed PCR results, which generally develops 5–6 days after infection (range, 1–14 days), consists of a myriad of respiratory symptoms with a climax of massive lung involvement, dyspnea, and death.^(1,2)^ Parallel to respiratory symptoms, SARS-CoV-2 infection can lead to a wide range of extrapulmonary, sensory, and neural complications, such as sudden onset olfactory and/or gustatory dysfunction,^(3,4)^ otological,^(5,6)^ and/or nonspecific symptoms, and long-term neurological complications.^(7)^

As pointed out by Jafari et al.,^(3)^ given that the suspicion of COVID-19 is mostly based on typical symptoms, patients with early onset sensory-neural manifestations, such as hearing loss, tinnitus, and/or dizziness/vertigo, may be misdiagnosed. To further complicate the early diagnosis of the audio-vestibular symptoms of these patients, many of them presented with very serious systemic conditions, with many co-morbidities and requiring prolonged hospitalizations in intensive care units (ICU) where they were sedated and aggressively treated, often with potentially ototoxic medications.

An additional barrier to early diagnosis was that most of these patients, even after leaving the ICU, remained convalesced for weeks in general hospitals without access to an appointment with an ear specialist or a full audiological workup.

Despite the difficulties in establishing a clear cause-and-consequence relationship, emerging evidence suggests a link between COVID-19 and the inner ear, adding a new dimension to the multifaceted impact of the virus on human health. With the pandemic progression, the nuances of this intriguing viral infection have been studied and understood more extensively.

Evidence that COVID-19 may cause not only hearing loss (especially sensorineural), but also tinnitus and a variety of balance symptoms has been substantiated by a series of meta-analyses that have unanimously pointed in this direction.

In one of the most robust meta-analyses which included 12 articles that met the eligibility criteria, the event rates for hearing loss, tinnitus, and dizziness were 3.1%, 4.5%, and 12.2%, respectively.^(3)^ Several studies have attempted to analyze the association between COVID-19 and ear symptoms more technically to examine the impact of the COVID pandemic on the incidence of new cases of SSD, but the results have been controversial.

Facing this new scenario, the close relationship between COVID-19 and ear symptoms went beyond empirical observations, small series of cases, and anecdotal case reports: these large population-based studies began to prove what otolaryngologists already suspected; that there was a clear outbreak of new and unexplained cases of deafness in a heterogeneous group of patients who shared a common denominator: having recently overcome an episode of COVID-19 infection.

## OBJECTIVE

The objective of this article was to describe a series of cases in which the association between SARS-CoV-2 infection and profound sensorineural hearing loss was temporarily present. The causal relationship could not be clearly established due to the clinical course of the disease, multiple clinical interactions, and prolonged use of potentially ototoxic medications.

## CLINICAL CASES

We must highlight that all patients included in this small series, according to their own reports (information obtained through interviews with their family members), did not present major hearing deficits prior to COVID-19 infection that were important enough to compromise their interpersonal communication and socialization.

## CASE 1

Male, 69 years old. In January 2022, the patient presented with severe acute respiratory failure following infection with COVID-19, requiring prolonged hospitalization ICU admission, and tracheostomy. During hospitalization, the patient developed acute sepsis with a pulmonary focus and was treated aggressively with large-spectrum antibiotics, including polymyxin B, amikacin, meropenem, vancomycin, and metronidazole.

When he was admitted to the hospital, he noticed bilateral hearing loss, which progressed quickly until its magnitude had a major impact on his communication.

**Figure 1:**
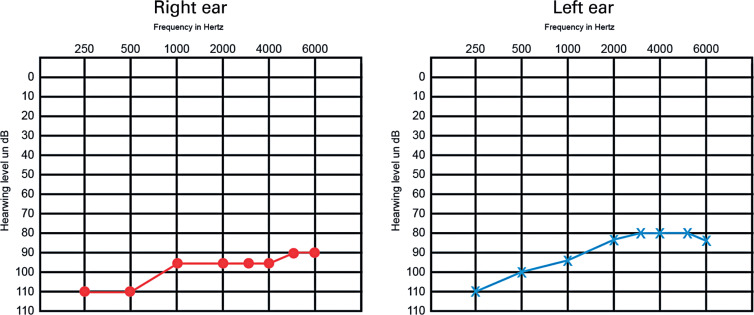
Audiogram patient 1

Shortly after being discharged, he was examined by an ear specialist. Clinical examination revealed no other major changes (the tracheostomy had already been removed); otoscopy was normal, and audiometry showed profound severe bilateral sensorineural hearing loss with no discrimination (Figure 1).

The patient was fit for powerful bilateral hearing-aids. After a trial period of 4 weeks, he reported no benefit from the use of hearing aids and was referred to the cochlear implant program.

## CASE 2

Female, 32 years old. A patient with chronic renal failure and no previous hearing complaints reported that two months earlier she was admitted to the hospital for the treatment of an infection in the hemodialysis catheter. During hospitalization, she underwent a serological diagnosis using PCR due to suspicions of a COVID-19 infection. Two days after the onset of symptoms, and without the use of any known ototoxic medication, she developed sudden bilateral hearing loss worsened by pulsatile tinnitus in both ears.

Bilateral symmetrical severe sensorineural hearing loss was confirmed through serial audiometry, even after the patient had been subjected to five intratympanic corticosteroid injections. The thresholds for 500, 1000, and 2000 Hz were 95, 90, and 85 dB, respectively, for the right ear and 90, 95, and 85 dB, respectively, for the left ear. The patient was fitted with powerful hearing aids with a fairly good response. This was the only patient in the group who did not receive ototoxic medication yet presented with the same type of hearing loss as the other patients.

**Table 1:** Summary of the clinical cases

	Case number						
	1	2	3	4	5	6	7
Gender	M	F	F	M	M	F	M
Age (years)	69	32	48	65	67	71	50
Respiratory Symptoms	yes	yes	yes	no	ICU	ICU	yes
Ototoxic Drugs	yes	no	yes	yes	yes	yes	yes
Hearing Loss in Hospital	yes	yes	yes	yes	yes	yes	yes
Vestibular Symptoms	no	no	no	no	no	no	yes
Average Hearing Loss (dB)	100	90	110	86	95	95	115
Hearing Aids	no	yes	no	yes	no	no	no
Cochlear Implants	yes	no	yes	no	yes	yes	yes

## CASE 3

Female, 48 years old. Patients with no previous hearing complaints reported hospital admission a year earlier for the treatment of a serious episode of respiratory infection due to COVID-19. She was admitted to the ICU for several weeks and noticed significant hearing loss in both ears upon discharge from the hospital. It could not be confirmed with certainty whether the hearing loss developed suddenly or progressively. She also reported continuous bilateral high-frequency and medium-intensity tinnitus. She did not have complete knowledge of the medications administered during her hospital stay, but was certain of having received polymyxin B and vancomycin. An ear, nose, and throat examination other than that for hearing loss and a decrease in the sense of smell were unremarkable. The hearing test showed profound bilateral severe sensorineural hearing loss with no discrimination.

The thresholds for 500, 1000, and 2000 Hz were 100, 110, and 120 dB, respectively, for the right ear and 90, 95, and 85 dB, respectively, for the left ear. The thresholds for 500, 1000, and 2000 Hz were 110, 115, and 120 dB, respectively, for the right ear and 100, 110, and 120 dB, respectively, for the left ear.

The patient was fit for powerful bilateral hearing-aids. After a trial period of three weeks, she reported no benefit from the use of hearing aids and was referred to the cochlear implant program.

## CASE 4

Male, 65 years old. Patient had a history of controlled arterial hypertension and acute myocardial infarction ten years ago. However, he did not report any hearing difficulties until his hospitalization for the treatment of a severe case of COVID-19 infection. He was hospitalized for five months during which he was on parenteral antibiotics, including amikacin, for at least a month. While convalescing at the hospital, he noticed progressive bilateral hearing loss that progressed within a few days. Due to his clinical condition, he could not be adequately evaluated by a specialist until a few weeks after discharge. A hearing test revealed severe bilateral sensorineural hearing loss with poor discrimination. The thresholds for 500, 1000, and 2000 Hz were 90, 90, and 80 dB, respectively, for the right ear, and 85, 80, and 70 dB, respectively, for the left ear.

Despite the magnitude of hearing loss, the patient was fitted with a hearing aid and was in a trial period to assess the benefits and needs of a cochlear implant.

## CASE 5

Male, 67 years old. In March 2021, the patient developed a severe COVID-19 infection accompanied by a prolonged ICU stay and required treatment with amikacin due to respiratory sepsis. He had no history of deafness or other otological disease; however, upon recovery from the infection, he reported bilateral hearing loss with very low auditory perception. Based on his medical history, he was diagnosed with kidney cancer during his COVID-19 hospitalization for which he underwent nephrectomy after recovery.

The hearing test was performed using hearing aids with a limited hearing gain. Therefore, cochlear implants were indicated. We chose to perform sequential bilateral surgery because of unfavorable clinical conditions for long-term surgery. The Implant was placed in the right ear in October, 2022. Before his left-ear implant was placed, six months after the first one, the patient suffered a cerebellar stroke. The patient is currently recovering without neurological sequelae and is expected to undergo an implant in his left ear soon.

The thresholds for 500, 1000, and 2000 Hz were 105, 90, and 90 dB, respectively, for the right ear, and 100, 95, and 90 dB, respectively, for the left ear.

## CASE 6

Female, 71 years old. A previously healthy patient was hospitalized in March 2021 because of severe COVID-19 infection. During the progression of the condition, clinical worsening was caused by pulmonary sepsis. Intensive care with invasive mechanical ventilation was required, and for 3 weeks, the patient was maintained on several IV antibiotics, but she was unable to say precisely whether any of them were ototoxic.

After recovering from the condition, the patient reported significant hearing loss with great difficulty in understanding and discriminating sounds. The thresholds for 500, 1000, and 2000 Hz were 95, 90, and 85 dB, respectively, for the right ear and 90, 95, and 85 dB, respectively, for the left ear. The thresholds for 500, 1000, and 2000 Hz were 105, 90, and 85 dB, respectively, for the right ear, and 100, 95, and 90 dB, respectively, for the left ear.

After hearing aid tests revealed insignificant hearing gain and limited discrimination of words and sentences, a cochlear implant was proposed.

## CASE 7

Male, 50 years old. The patient had no hearing or balance complaints until June 2021, when he experienced a rapidly progressive COVID-19 infection. He received numerous antibiotic treatments, including amikacin. He developed acute kidney failure and underwent weekly dialysis sessions since then. On admission, he noticed progressive deterioration in hearing ability, muscle weakness, and severe imbalance. As he was admitted to a low-complexity hospital, he sought ear, nose, and throat services several weeks after discharge and attended a consultation with bilateral hearing aids with very limited gain. On examination, in parallel with profound bilateral deafness, the patient presented with marked ataxia and imbalance, possibly due to bilateral labyrinthopathy. The thresholds for 500, 1000, and 2000 Hz were 110, 115, and 120 dB, respectively, for the right ear, and 100, 110, and 120 dB, respectively, for the left ear.

Based on his clinical history, a bilateral implant was indicated, which was initially performed on the right ear. The patient showed satisfactory evolution with improved hearing gain and balance after vestibular rehabilitation sessions while waiting for the implant in the left ear.

## DISCUSSION

This small case series suggests a possible association between Covid infection and the development of ear symptoms. However, the cause-and-consequence relationships may be affected by a series of associated events. For example, of the seven patients, five presented with extremely critical general condition. All patients spent long hospital journeys and lengthy periods in the ICU, and used potentially ototoxic medications during hospitalization.

In contrast, an outbreak of sudden unilateral hearing loss (SSD) in non-critical Covid patients was observed during the same period. These patients were not severely ill or taking ototoxic medications.

Saniasiaya et al. conducted a study showing that the incidence of SSNHL and combined acute cochlear-vestibular involvement was significantly higher during the COVID-19 pandemic than in previous periods, with a more severe clinical presentation on pure-tone audiometry.^(8)^ Another study by Cooper et al found an increased incidence of SSNHL during the COVID-19 epidemic.^(9)^ A large tertiary hospital in China also found an increase in SSNHL visits to outpatient and emergency departments during the COVID-19 pandemic.^(10)^

Pointing in the opposite direction, at least two meta-analyses and one systematic review failed to clearly establish the link between Covid infection and the emergence of audio-vestibular symptoms, unanimously suggesting the need for new, better-controlled studies to avoid bias and confounding factors.^(3,5,11)^

Even so, since the beginning of the pandemic, we have noticed a sharp increase in the number of patients who developed ear symptoms (during the disease or shortly after), such as sudden unilateral hearing loss, tinnitus, or even progression of well-established hearing loss (which previously did not affect interpersonal communication).

It is also important to mention that cases of SSD in non-hospitalized individuals were clearly classified as a new event and promptly reported as such. On the other hand, mild to moderate bilateral hearing deficits were not flagrant because they often affected an older population without a previous hearing test for comparison. Interestingly, many of these patients sought medical help when they noticed a decrease in their communication skills after the widespread use of masks and the consequent loss of facial cues.

However, several clinical studies^(11–15)^ (later pooled into large meta-analyses) suggest that these initial empirical observations could represent the tip of the iceberg of a real clinical phenomenon. Emerging evidence suggests a potential link between COVID-19 and hearing loss, adding a new dimension to the multifaceted impact of the virus on human health. Maharaj et al. pooled 28 patients with otological dysfunction, all of whom had sensorineural hearing loss, of which 27 underwent audiograms. Three patients presented with vestibular symptoms.^(15)^ As mentioned previously, Jafari et al. conducted a meta-analysis based on four studies and concluded that the occurrence of hearing loss in all confirmed COVID-19 patients was 3.1%.^(3)^

It would be interesting to understand audiogram findings in these patients. Patients usually present with some degree of simultaneous bilateral sensorineural deafness, which can be mild or severe.^(7,12)^ Simultaneous sensorineural hearing loss is rare yet frequent in post-COVID-19 patients. In a revision paper, it was found that 20.6%, 6.3%, 31.7%, 4.8%, 11.1%, 20.6%, and 4.8% of patients had mild, mild-to-moderate, moderate, moderate-to-severe, severe, severe-to-profound, and profound hearing loss, respectively.^(2)^

Most of the reviewed studies did not attempt to analyze the changes in the inner ear caused by COVID-19 infection. However, Yamada et al. suggested three possible mechanisms of hearing loss: thrombosis leading to cochlear vascular obstruction, viral infection, and cochlear inflammation.^(14)^

A series of other pathophysiological theories seek to explain the damage caused by COVID to the audio-vestibular system.

Viral Invasion and the Inner Ear: The SARS-CoV-2 primarily targets the respiratory system; however, recent studies have proposed that it may also invade other organs, including the inner ear; The delicate structures of the inner ear, which are responsible for hearing and balance, are susceptible to viral infections, leading to inflammation and damage.

Inflammatory Response and Auditory System: COVID-19 triggers a robust immune response in the body, characterized by inflammation. The proximity of the inner ear to the central nervous system makes it vulnerable to inflammatory cascades, potentially damaging the auditory nerve and associated structures. This inflammatory response can manifest as temporary or permanent hearing loss.

Oxidative Stress and Hearing Impairment: COVID-19-induced oxidative stress is another factor implicated in development of hearing loss. Oxidative stress occurs when there is an imbalance between the production of free radicals and the body's ability to neutralize them. This imbalance can lead to cellular damage in the auditory system and contribute to hearing impairment.

Complications and Long COVID: Individuals with severe COVID-19 cases or those experiencing prolonged symptoms, often referred to as "long COVID," may be at a higher risk of developing complications, including hearing issues. The extended inflammatory response and lingering effects of the virus may contribute to persistent auditory deficits.

Ototoxic Medications and COVID Treatment: Medications used to manage severe COVID-19 cases, such as certain antibiotics and antivirals, have been associated with potential ototoxic harmful effects on the ear. These medications can exacerbate the existing hearing issues or contribute to the development of new auditory problems in COVID-19 patients.

Pre-existing Conditions and Hearing Loss: COVID-19 disproportionately affects individuals with pre-existing health conditions, some of which are associated with hearing loss. Conditions such as diabetes and cardiovascular disease, prevalent among COVID-19 patients, can independently contribute to auditory problems by creating a complex interplay of factors.

Vestibular changes have also been described by Pazdro-Zastawny et al., who stated that, in some cases, they are central, indicating a possible neurotropic effect of the virus.^(13)^ Yamada et al., studied magnetic resonance images and found increased contrast in the cochlea, vestibule, and lateral and superior semicircular canals in each ear .^(14)^

It has been shown that the neuroinvasion driven by SARS-CoV-2 is associated with the angiotensin-converting enzyme 2 (ACE2) mechanism, as a functional receptor for the virus.^(12)^ This enzyme receptor is commonly found in the lung type 2 alveoli. It is expressed in many cell types, including glial cells and neurons, and can cause neurological involvement through direct or indirect mechanisms.^(11,13)^

Cooper et al., in a study that included all COVID-19 patients, established the relationships between hyperinsulinemia, magnesium, vitamin D, and thrombosis. The authors suggested that hyperinsulinemia promotes magnesium depletion by increasing renal excretion and reducing intracellular magnesium, which lowers vitamin D levels. Hyperinsulinemia disturbs fibrinolysis and provokes an increase in thrombosis and emboli, which have been detected in postmortem findings of COVID-19 cases.^(9)^

Although our series consisted of only seven cases, it is interesting to note that we were able to establish a certain pattern.

1. Average age 57 years (32-71).
2. Not dependent on gender.
3. No consistent auditory complaints prior to infection.
4. At least one patient reported associated tinnitus (Case #2) and severe imbalance (Case #3).
5. Severe COVID-19 requiring long hospital stays with variable periods in intensive care units.
6. All but one (case #7) had previous important comorbidities.

A. Association with the use of potentially ototoxic medications (except in case 3); B. Everyone already had Significant deafness at the time of hospital discharge; C. The loss was sensorineural, bilateral, and gravitated between severe and profound degrees. These observations are important for audiological follow-up and rehabilitation strategies.

## CONCLUSION

Our study demonstrates that the world continues to grapple with the far-reaching consequences of COVID-19, and it becomes increasingly evident that the impact extends beyond immediate respiratory distress. Whether the pathophysiological mechanism of these complications is directly or indirectly related to the virus remains to be clearly demonstrated; however, the potential relationship between COVID-19 and hearing loss underscores the need for comprehensive healthcare strategies that consider the diverse manifestations of the virus.

Further research is essential to unravel the intricate mechanisms linking COVID-19 to hearing impairment and develop targeted interventions. Healthcare professionals should remain vigilant in monitoring and addressing auditory symptoms in COVID-19 patients, emphasizing the importance of a holistic approach to post-COVID care.

## Data Availability

The underlying content is contained within the manuscript and the content will be made available upon the article's publication.

## References

[1] Fancello V, Hatzopoulos S, Corazzi V, Bianchini C, Skarżyńska MB, Pelucchi S (2021). SARS-CoV-2 (COVID-19) and audio-vestibular disorders. Int J Immunopathol Pharmacol.

[2] Fancello V, Fancello G, Hatzopoulos S, Bianchini C, Stomeo F, Pelucchi S (2022). Sensorineural Hearing Loss Post-COVID-19 Infection: An Update. Audiol Res.

[3] Jafari Z, Kolb BE, Mohajerani MH (2022). Hearing Loss, Tinnitus, and Dizziness in COVID-19: A Systematic Review and Meta-Analysis. Can J Neurol Sci.

[4] Lee SW, De Chua WD, Yuen HW (2022). Relationship between SARS-CoV-2 and hearing loss. Proc Singapore Healthcare.

[5] Frosolini A, Franz L, Daloiso A, de Filippis C, Marioni G (2022). Sudden Sensorineural Hearing Loss in the COVID-19 Pandemic: A Systematic Review and Meta-Analysis. Diagnostics (Basel).

[6] Mehraeen E, Afzalian A, Afsahi AM, Shahidi R, Fakhfouri A, Karimi K (2023). Hearing loss and COVID-19: an umbrella review. Eur Arch Otorhinolaryngol.

[7] Bozdemir K, Çallıoğlu EE, İslamoğlu Y, Ercan MK, Eser F, Özdem B (2024). Evaluation of the effects of COVID-19 on cochleovestibular system with audiovestibular tests. Ear Nose Throat J.

[8] Saniasiaya J (2021). Hearing Loss in SARS-CoV-2: What Do We Know?. Ear Nose Throat J.

[9] Cooper ID, Crofts CA, DiNicolantonio JJ, Malhotra A, Elliott B, Kyriakidou Y (2020). Relationships between hyperinsulinaemia, magnesium, vitamin D, thrombosis and COVID-19: rationale for clinical management. Open Heart.

[10] Jin L, Fan K, Tan S, Liu S, Wang Y, Yu S (2021). Analysis of the characteristics of outpatient and emergency diseases in the department of otolaryngology during the "COVID-19" pandemic. Sci Prog.

[11] Meng X, Wang J, Sun J, Zhu K (2022). COVID-19 and Sudden Sensorineural Hearing Loss: A Systematic Review. Front Neurol.

[12] Chirakkal P, Al Hail AN, Zada N, Vijayakumar DS (2021). COVID-19 and Tinnitus. Ear Nose Throat J.

[13] Pazdro-Zastawny K, Dorobisz K, Misiak P, Kruk-Krzemień A, Zatoński T (2022). Vestibular disorders in patients after COVID-19 infection. Front Neurol.

[14] Yamada S, Kita J, Shinmura D, Nakamura Y, Sahara S, Misawa K (2022). Update on Findings about Sudden Sensorineural Hearing Loss and Insight into Its Pathogenesis. J Clin Med.

[15] Maharaj S, Bello Alvarez M, Mungul S, Hari K (2020). Otologic dysfunction in patients with COVID-19: A systematic review. Laryngoscope Investig Otolaryngol.

